# Impact of annotation imperfections and auto-curation for deep learning-based organ-at-risk segmentation

**DOI:** 10.1016/j.phro.2024.100684

**Published:** 2024-12-04

**Authors:** Victor I.J. Strijbis, O.J. Gurney-Champion, Berend J. Slotman, Wilko F.A.R. Verbakel

**Affiliations:** a Amsterdam UMC location Vrije Universiteit Amsterdam, Department of Radiation Oncology, De Boelelaan 1117, Amsterdam, the Netherlands; b Cancer Center Amsterdam, Cancer Treatment and Quality of Life, Amsterdam, the Netherlands; c Amsterdam UMC location University of Amsterdam, Department of Radiology and Nuclear Medicine, Meibergdreef 9, Amsterdam, Netherlands; d Cancer Center Amsterdam, Imaging and Biomarkers, Amsterdam, the Netherlands; e Varian Medical Systems, a Siemens Healthineers Company, Palo Alto, USA

**Keywords:** Head-and-neck cancer, Radiotherapy, Deep learning, Segmentation, Automated curation, Data quality

## Abstract

**Background and purpose:**

Segmentation imperfections (noise) in radiotherapy organ-at-risk segmentation naturally arise from specialist experience and image quality. Using clinical contours can result in sub-optimal convolutional neural network (CNN) training and performance, but manual curation is costly. We address the impact of simulated and clinical segmentation noise on CNN parotid gland (PG) segmentation performance and provide proof-of-concept for an easily implemented auto-curation countermeasure.

**Methods and Materials:**

The impact of segmentation imperfections was investigated by simulating noise in clean, high-quality segmentations. Curation efficacy was tested by removing lowest-scoring Dice similarity coefficient (DSC) cases early during CNN training, both in simulated (5-fold) and clinical (10-fold) settings, using our full radiotherapy clinical cohort (RTCC; N = 1750 individual PGs). Statistical significance was assessed using Bonferroni-corrected Wilcoxon signed-rank tests. Curation efficacies were evaluated using DSC and mean surface distance (MSD) on in-distribution and out-of-distribution data and visual inspection.

**Results:**

The curation step correctly removed median(range) 98(90–100)% of corrupted segmentations and restored the majority (1.2 %/1.3 %) of DSC lost from training with 30 % corrupted segmentations. This effect was masked when using typical (non-curated) validation data. In RTCC, 20 % curation showed improved model generalizability which significantly improved out-of-distribution DSC and MSD (p < 1.0e-12, p < 1.0e-6). Improved consistency was observed in particularly the medial and anterior lobes.

**Conclusions:**

Up to 30% case removal, the curation benefit outweighed the training variance lost through curation. Considering the notable ease of implementation, high sensitivity in simulations and performance gains already at lower curation fractions, as a conservative middle ground, we recommend 15% curation of training cases when training CNNs using clinical PG contours.

## 1 Introduction

Radiotherapy target and organ-at-risk (OAR) segmentation is a complex, resource-intensive and semi-subjective process that requires domain expertise and suffers from inter- and intra-observer variabilities [1], [2], [3]. Despite consensus guidelines [4], [5], errors and inconsistencies in segmentation datasets persist [6].

Convolutional neural networks (CNNs) have emerged to reduce clinical burdens and improve segmentation standardization but require large amounts of data for adequate training. Using clinically available segmentations can result in sub-optimal training [7], [8], [9], [10], [11], [12], when segmentations contain substantial noise. Apart from extensive manual correction [13], [14], [15], several engineering solutions exist to make CNNs more robust to classification label noise [13], [16], [17], [18], [19], [20], but solutions for segmentation are limited [12].

Methods that address how noisy segmentations affect CNNs typically directly apply techniques used for removing label noise in classification. This assumes independent and identically distributed noise (errors randomly occur anywhere within the segmentation) [21]. This is unrealistic, since manual segmentation (using brushes) typically introduces errors near structure boundaries with both variance (random deviations) and bias (e.g., missing structure segments like accessory glands [22], systematic over-/under-segmentation) components [23]. Effects of these error modes on CNN training and performance are variable [10] and both may require different approaches to ameliorate [21].

Due to its relevance for dose planning, the parotid gland (PG) is among the primarily included OARs for head-and-neck cancer (HNC) and has sufficient shape/size variability, making it a well-understood paradigm to study the impact of segmentation noise on auto-segmentation accuracy.

We address how PG segmentation bias and variance influence CNN training. Additionally, we propose an easy-to-implement auto-curation method to make CNNs more robust and flexible when handling segmentation bias, which can be practically used to further autonomize segmentation among institutes and reduce review time of automated segmentations.

## 2 Methods & Materials

Here, curation means removal of cases for which CNNs perform poorly during training. We first established baseline performance on in-house PG segmentations using cross-validation. Then, using a subset of known clean segmentations, we studied the impact of imperfect segmentations by manually adding corruptions and determined curation efficacy in a large, clinical cohort.

### 2.1 Data

The clinical dataset consisted of 1925 PG (1563 SMG; post-hoc) segmentations paired with 3D planning CTs acquired using one scanner during the standard clinical protocol for a HNC patient cohort treated between 2009 and 2021 and is named radiotherapy clinical cohort (RTCC). Segmentations were made by one out of 18 radiation oncologists, who were assumed to follow consensus guidelines [4]. The American Association for Medical Physics (AAPM) HNC segmentation challenge dataset [24] was included as external (out-of-distribution) data. Dataset (pre-processing) details are supplemented (SMA.1). Due to this study’s retrospective nature, not including tests or different treatments on human individuals and the use of pseudonymized data, our Medical Ethics Committee concluded that the Medical Research involving Human Subjects Act (WMO) does not apply and has exempted this study for official approval.

### 2.2 Dataset curation

**Fig. 1:**
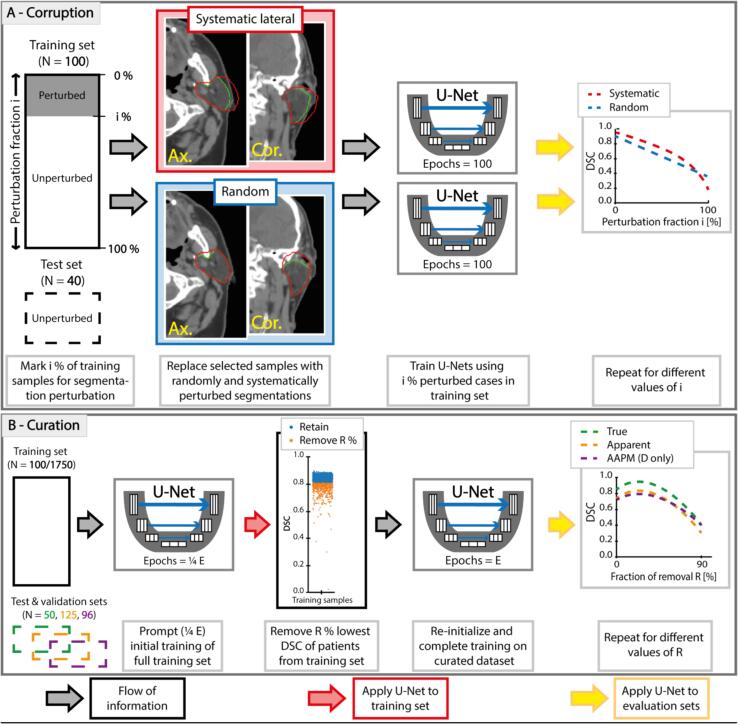
Corruption and curation schematic overview. A: Examples of data corruption (red contour) versus non-corrupted clinical segmentation (green contour) are shown in axial and coronal planes. The systematic perturbation was always lateral. The random dilation example shown was a caudo-cranial expansion but could be any direction. Models are evaluated using clean (non-corrupted) test cases from RTCC-Q1 and from AAPM. B: For curation, after prompt (25% of epochs) training, the CNN is applied to input training data, before reinitializing and fully training the network without the lowest DSC cases and evaluating on independent validation data. Models are evaluated using DSC. AAPM data are used only for validation of clinically representative data (experiment series D). Abbreviations: i: fraction of perturbed data; DSC: Dice similarity coefficient; R: fraction of removal. AAPM: American Association of Medical Physics. (For interpretation of the references to colour in this figure legend, the reader is referred to the web version of this article.)

CNNs initially learn dominant patterns among correct data samples, before learning details from a minority of corrupted segmentations [16]. Hence, we hypothesize that cases with corrupt segmentations have poorer performance than with correct segmentations, especially early during training. Therefore, we utilize a sample selection method as a simple curation method (Fig. 1A). First, CNNs were trained for 25 % of epochs required for full training. Details are supplemented (SMA.2). Then, CNNs were evaluated on all input training data. The poorest performing cases were removed from training with the removed amount being a tunable parameter. Subsequently, networks were re-initialized and training was restarted from scratch for full training on curated data.

### 2.3 Experimental outline

We analyzed (A) baseline performance by cross-validation; (B) corruption simulation; (C) curation of simulated corruptions and (D) curation of clinical data (supplementary methods SMA.3). Experiment series B and C(4–7) used perturbation operations to obtain corrupted segmentations (Fig. 1A). Experiment series C(2,3,5,7) and D relied on our curation operation (Fig. 1B) to investigate to what extent simulated corruptions were undone, and determine efficacy in clinical data.

#### 2.3.1 Cross-validation

To investigate baseline performance in non-curated data and establish a trustworthy dataset for segmentation corruption and curation experiments, we performed 5-fold cross-validation using train/val/evaluation (1365/175/385) RTCC sample splits. Thereby, for all cases, CNN-segmentations and DSC-agreements were determined. To estimate the severity of segmentation imperfections in our datasets, two specialists (consensus) reviewed 40/25 cases from the lowest/highest DSC quartiles, respectively. Each segmentation was scored for quality, depending on parameters: segmentation excludes (considerable portions of) a: medial lobe; b: anterior lobe; c: caudal tissue; d: dorsal tissue; or e: includes substantial bone/skin/air. Quality scores assigned were: 1: defective (≥three shortcomings with poor overall quality); 2: poor (two shortcomings); 3: insufficient (one shortcoming); 4: satisfactory (1–2 mm inaccuracies); 5: good (no noteworthy shortcomings). To mitigate the risk of removing difficult but correct cases, we suppose only severe errors (<4) should be excluded from “curated datasets” for CNN training.

#### 2.3.2 Segmentation corruption

To test how corrupted reference segmentations influenced model performance, models were trained using segmentations that were actively corrupted in varying amounts. To minimize confounders from segmentation noise in baseline samples, 180 (100/40/40 train/val/test) cases were first randomly selected from the highest-scoring quartile (Q1) of the initial cross-validation. Multiple models, each containing 5 % increasing amounts of either systematic or random corruptions (Fig. 1A), were then trained and evaluated using identical train/val/test allocations, resulting in 42 models. To emphasize how learning with faulty labels affected “true” performance, training performances were displayed.

Inspired by clinical observations that included skin/bone/air (likely driven by lateral fan-out during clinical contouring; SMB.1), segmentation corruptions were introduced by dilating/eroding existing Q1 segmentations. In systematic corruptions, clinical segmentations were laterally dilated (10 voxels; 1.0 cm along the sagittal (L/R)-axis). In random corruptions, segmentations are dilated or eroded (p = 1/2), in any Cartesian direction (p = 1/6). Dilation magnitudes were the same as in systematic corruptions, but erosions were 20 % smaller to compensate for the size-sensitivity of DSC-loss. The corruption extent was chosen such that the median DSC of 100 %-perturbed training data matched the median DSC of the lowest-scoring DSC quartile (Q4).

#### 2.3.3 Curation of simulated corruptions

To assess curation efficacy in data with known corruption severity (Fig. 1A), we evaluated auto-curation on datasets with systematically (red) and randomly (blue) corrupted segmentations, generated in our controlled setting (B). For this, seven experimental sub-categories were run. Three experiments used clean training data: for (1) baseline without patient removal; (2) 30 % random data removal, illustrating the performance decrease from reduced training data; (3) 30 % data removal through auto-curation, estimating the performance decrease in clean data. Following, four experiments were done using 30 % laterally (4–5) or randomly (6–7) dilated training segmentations: (4,6) without and (5,7) with 30 % auto-curation to estimate curation efficacy. The 30 % corruption fraction was chosen as it was previously indicated as a critical point for classification labeling errors [20], [25]. All experiments (1–7) were repeated 5-fold to estimate the spread of models trained with different random seeds.

#### 2.3.4 Clinical curation

Four experiments assessed curation efficacy in clinical data. Using all RTCC training cases, (1) auto-curation and (2) random removal were performed with rates between 0–90 %. Subsequently, models with the highest curation efficacy across all test/validation sets were used to visually inspect AAPM segmentations and were compared to non-curated model AAPM segmentations. To evaluate statistical variation, this was repeated using repeated random sub-sampling (RRSS) of 100 cases randomly sampled from RTCC (3–4). The full RTCC analysis was repeated 10-fold only for the highest curation efficacy fraction. The highest mean class probability (before sigmoid layer) from all models was used to obtain ensemble contours to study how ensembles improved individual models and were affected by curation. As validation in other OARs, clinical curation was applied post-hoc to SMG.

### 2.4 Curation error analysis

Auto-curation methods that work by sample removal risk excluding potentially informative samples from training [26], [27]. Therefore, we randomly sampled and reviewed cases removed by curation. Finally, curation agreement was computed as the average overlap of curated cases among all (combinations of) iterations.

### 2.5 Model

All models used identical hyper-parameters and architectures (3D U-Net) [28], adapted for using 4 depth layers, 32 input-layer filters (3x3x3 voxels) and filters doubling at each depth layer. Batch-norm was applied after non-linear activations. Model training specifics are supplemented (SMA.4).

### 2.6 Clinical validation & test

To evaluate auto-curation efficacy in clinical data, it is essential that test sets contain minimal segmentation noise [21]. Therefore, different datasets were used to evaluate curation in clinical data. We refer to performance evaluations using clinical data with minimal amounts of segmentation imperfections as true-performance test sets and data that mimics non-curated training distributions as apparent-performance validation sets. In clinical curation experiments (D), true-performance sets consisted of in-house data obtained by manually reviewing random RTCC cases. If substantial imperfections were absent, cases were moved from training to the true-performance set, until 50 cases were approved. Following, to construct the apparent-performance set, 125 additional cases were randomly sampled from RTCC without manual review. AAPM data was used as independent test data [24]. In experiments of corruption impact and curation of simulated corruptions (B-C), the in-house test data came from clean Q1-cases and is therefore essentially a true-performance test set. The validation set was actively corrupted to represent apparent performance.

### 2.7 Evaluation

Performances were measured with DSC, (maximum) Hausdorff distance (HD) and mean surface distance (MSD) between CNN– and reference segmentations, except for experiments A-B-C, where HD/MSD were omitted. Curation efficacy was defined as curated minus non-curated DSC, and sensitivity as the fraction of correctly removed (quality < 4) corrupted contours.

### 2.8 Statistical analysis

Since DSC is not normally distributed, Wilcoxon signed-rank tests with Bonferroni-correction were used for 27 comparisons. This correction was enforced by adjusting the significance threshold (alpha-value = 0.05) to 1.9e-3. Statistical tests were performed only when indicated in text/figures. To ensure independence of data points in experiments with repeated tests, the resulting distribution of medians over all iterations was tested for significance.

### 2.9 Post-hoc SMG analysis

To investigate how curation translates to other datasets, the extent of segmentation inconsistencies of 1704 (1363/171/170 train/val/test split) in-house SMGs was estimated in 5 experiment iterations. Since AAPM data deviated substantially from contouring guidelines, they were unsuitable for benchmarking out-of-distribution curation efficacy and were included for training as curatable “erroneous” segmentations (rate = 5 %), instead. Curated cases were quality-scored and efficacy on in-house data was determined. Details are supplemented (SMA.2–4).

## 3 Results

### 3.1 Baseline (A)

Median[IQR] DSC of the cross-validation was 0.867[0.835–0.889], with a Q4-median of 0.803. Visual inspection of Q1-contours confirmed no substantial shortcomings, while imperfections in Q4 were much larger and typically missed (segments of) anterior/medial lobes or included substantial portions of skin/bone/air (SMB.1). Occurrence rates of quality scores (1–5) were (0 %-0%-0%–32 %-68 %)/(15 %-25 %-35 %–22.5 %-2.5 %) for Q1/Q4 (N = 25/40), respectively.

### 3.2 Segmentation corruption (B)

**Fig. 2:**
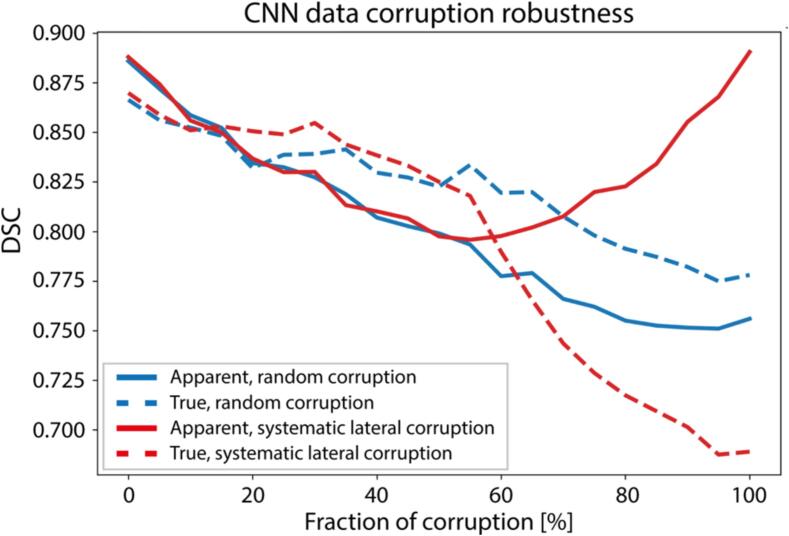
Impact of simulated corruptions. Impact of increasing fractions of random (blue) or systematic lateral (red) training data corruptions on DSC, evaluated on clean test data (dashed, true performance) and on data with corruptions as in training (solid line, train performance). Abbreviations: CNN: Convolutional neural network; DSC: Dice similarity coefficient. (For interpretation of the references to colour in this figure legend, the reader is referred to the web version of this article.)

Corrupted segmentations were created with median(range) agreements (DSC) of 0.778(0.720–0.834) and 0.795(0.720–0.836) for systematic lateral and random corruptions, respectively. True-performance decreased more for random than systematic noise if <50 % of training segmentations were corrupted, whereas this trend reversed when corruption fractions increased (Fig. 2). Hereafter, CNNs learned and reproduced systematic noise more rigidly than random noise (Fig. 2, solid red).

### 3.3 Curation of corrupted contours (C)

**Fig. 3:**
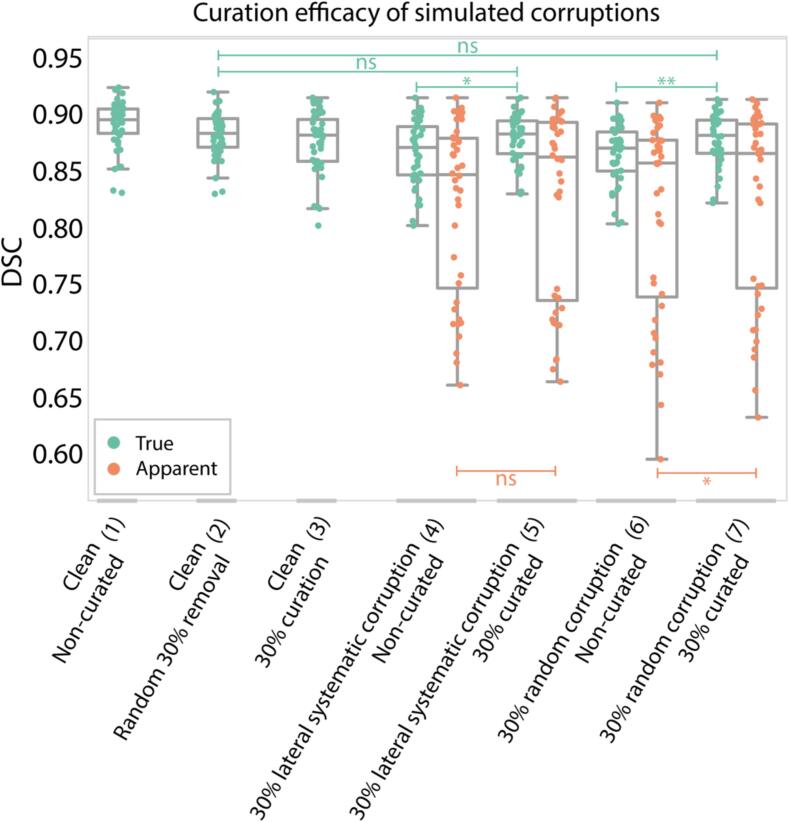
Curation in controlled setting. Comparison of DSC distributions between different data corruption and curation conditions. The decrease in performance from (1) to (2) indicates model deterioration due to decreased sample size. The decrease from (2) to (3) indicates the effect of model curation on detected corrupted samples. Significance bars indicate conditions tested for statistical significance. Significance levels: *: p-value < 1e-3; **: p-value < 1e-6. Abbreviations: DSC: Dice similarity coefficient; ns: not significant.

Curation operations recovered on average 0.116/0.127 (absolute DSC) performance lost by corruption and were statistically indistinguishable from random removal (Fig. 3). For 30 % systematic/random corruptions, corrupted segmentations were correctly curated in median(range) 97(96.7–100)/100(90–100)% of cases. Curation significantly increased median[IQR] true-performance DSC from 0.872[0.848–0.891] to 0.884[0.867–0.896] (p = 5.8e-6) for systematic and from 0.872[0.851–0.886] to 0.883[0.867–0.896] (p = 1.0e-7) for random corruptions. Additionally, considering the discontinuity of apparent-performance data points, CNNs trained on curated data were more consistent than CNNs trained on non-curated data (Fig. 3.5;3.7).

### 3.4 Clinical curation (D)

**Fig. 4:**
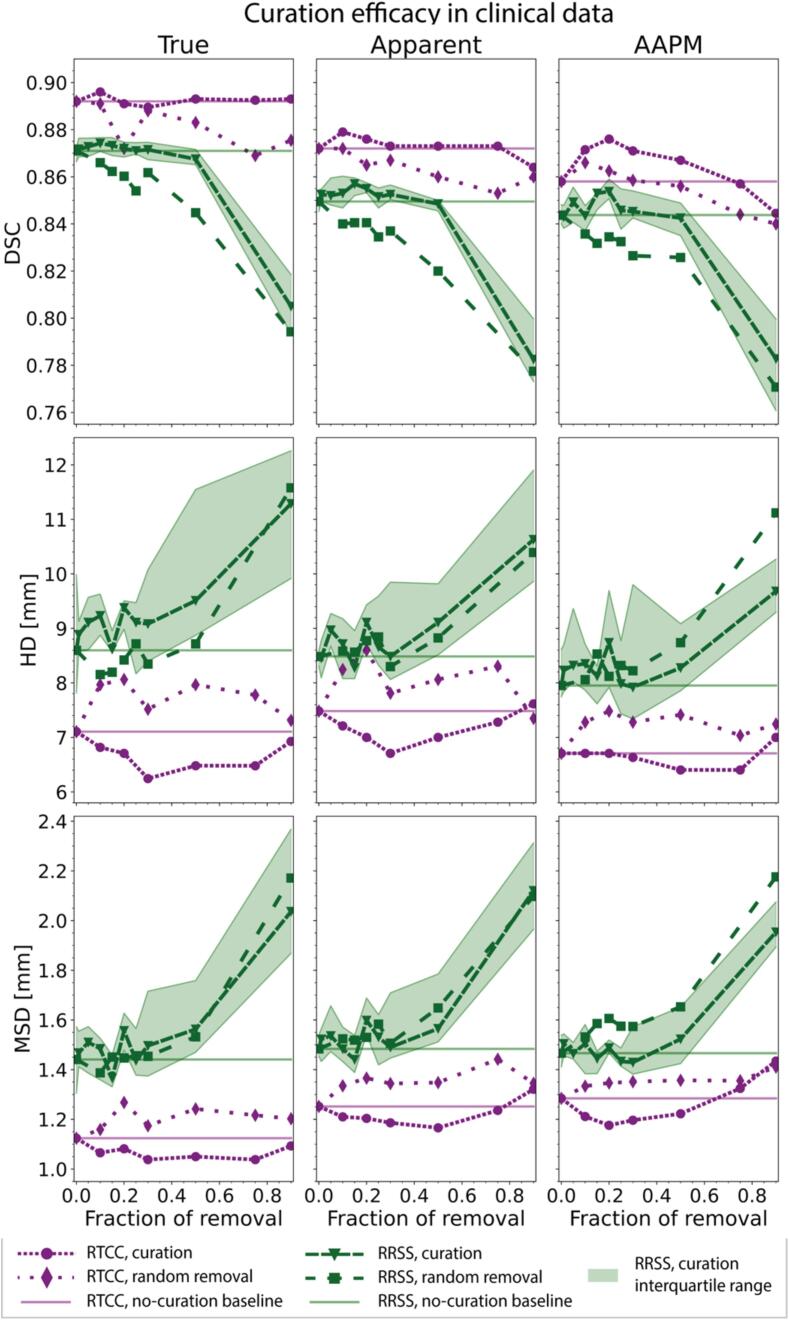
Clinical curation efficacy. Clinical curation experiment series overview, with validation sets of true, apparent and external AAPM performance. Green and purple indicate RRSS and RTCC experiments, respectively. Displayed sub-sampling (green) results were median values from 10 repetitions. IQR was computed for both the random removal and removal by curation in RRSS, but was omitted from visualization for random removal for graph readability purposes. Abbreviations: AAPM: American Association of Medical Physics (out-of-distribution); DSC: Dice similarity coefficient; HD: Hausdorff distance; MSD: mean surface distance. RTCC: radiotherapy clinical cohort; RRSS: repeated random sub-sampling; IQR: interquartile range. (For interpretation of the references to colour in this figure legend, the reader is referred to the web version of this article.)

**Fig. 5:**
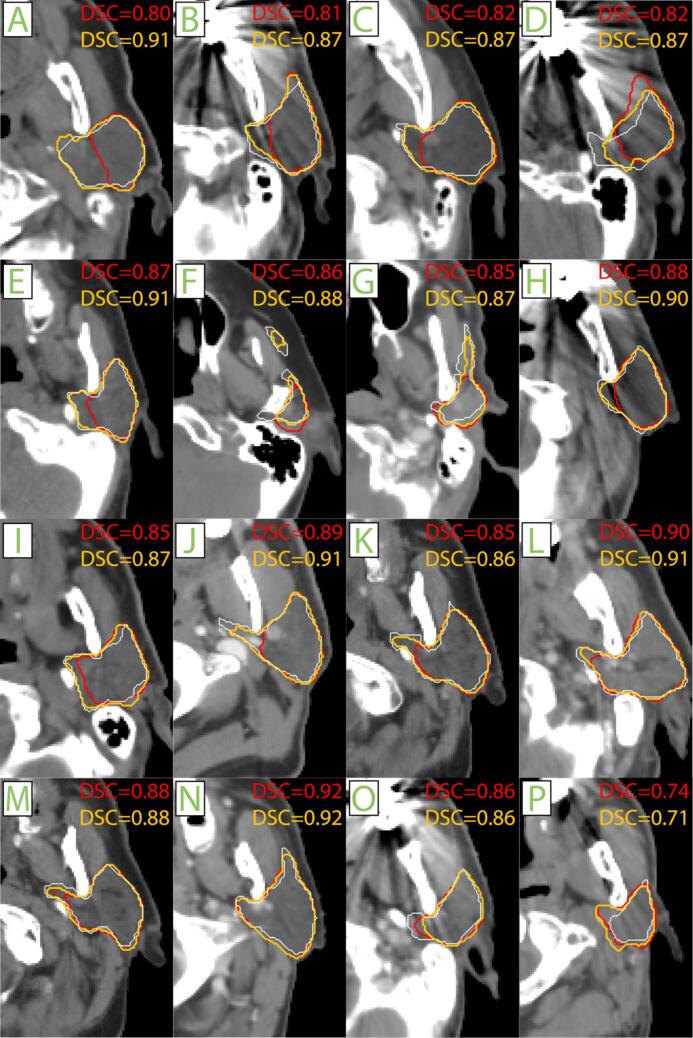
Visual decomposition of clinical curation effect. The 20% curated RTCC model (orange) typically showed better medial lobe agreement with the manual reference (white) than the non-curated RTCC model (red) in out-of-distribution data. For each case, the curation efficacy was computed and rank-ordered. This figure visualizes every 6th sample with respect to increasing curation efficacy. Abbreviations: DSC: Dice similarity coefficient. (For interpretation of the references to colour in this figure legend, the reader is referred to the web version of this article.)

In singular CNN training, pre-curation, true, apparent and AAPM median [IQR] DSCs were 0.892[0.869–0.906], 0.872[0.835–0.896], and 0.858[0.827–0.880], respectively (Fig. 4; solid purple). No evident changes in true/apparent performance were observed from curation. However, AAPM performance substantially improved with curation rates ≤50 %, with a maximum median[IQR] DSC increase of 1.9 % to 0.877[0.855–0.897] (p < 1.0e-9), and MSD improvement from 1.29[1.16–1.51] to 1.18[1.07–1.34] mm (p < 1.0e-9; Fig. 4; dense purple) at 20 % removal. In apparent and AAPM data, DSC/MSD performance initially improved, and deteriorated compared to the non-curation baseline when ≥75 % cases were discarded, whereas true DSC/MSD did not deteriorate from any curation fraction. Visual comparison of AAPM segmentations, generated using 0 %- and 20 %-curated models, showed that curated models were more consistent and accurate particularly in medial (Fig. 5A–E,H,I–M), and anterior lobes (Fig. 5F–G). Manual review of random Q4- and Q1-cases showed that curation successfully removed 83 % of training samples below the acceptance threshold(<4).

**Fig. 6:**
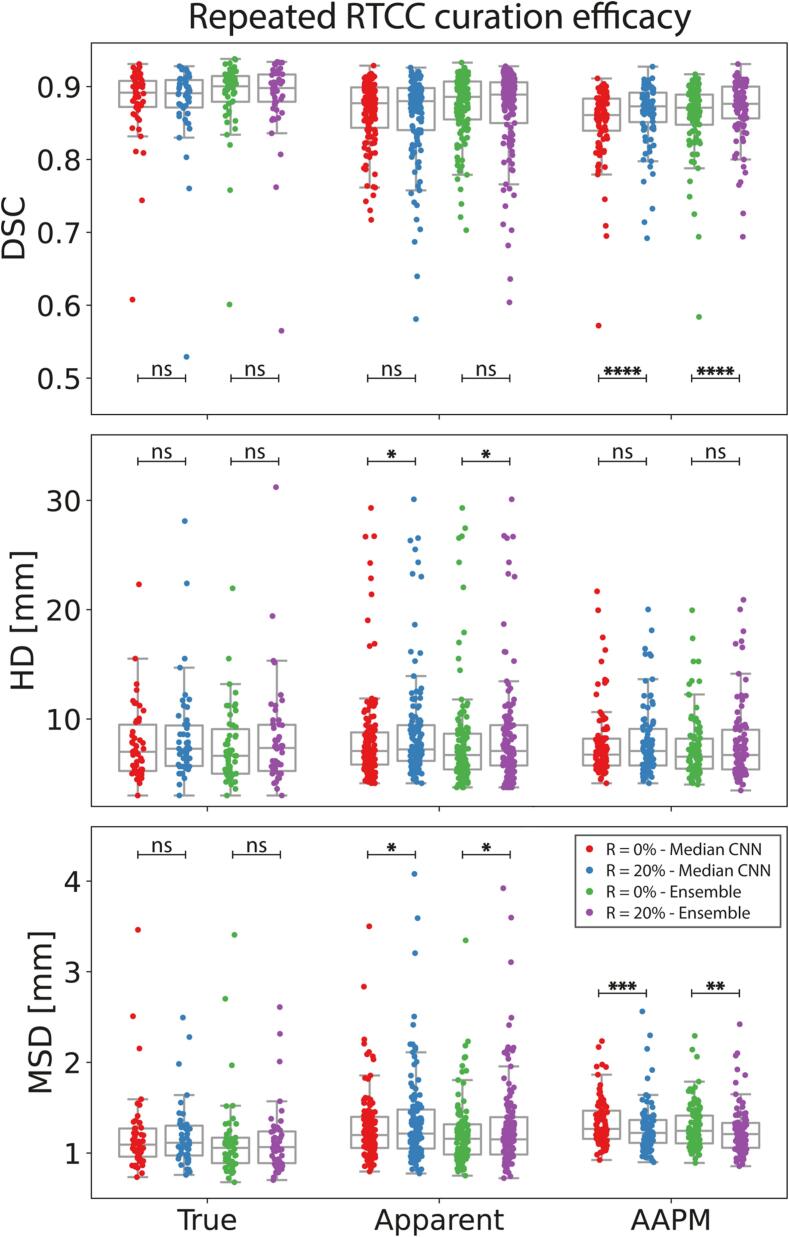
Ensembles and curation. 20 % curation significantly improved out-of-distribution DSC and MSD performance in both individual (median) CNNs and CNN ensembles. For the median CNN, data points were obtained by taking the median metric over 10 model repetitions for each case. Significance levels: *: p-value < 1e-3; **: p-value < 1e-6; ***: p-value < 1e-9; ****: p-value < 1e-12; ns: not significant. Abbreviations: AAPM: American Association of Medical Physics; DSC: Dice similarity coefficient; HD: Hausdorff distance; MSD: mean surface distance; CNN: convolutional neural network.

When both curation conditions (0 %/20 %) were repeated 10-folds (Fig. 6; Median CNN), curation improved median[IQR] AAPM DSC from 0.861[0.840–0.884] to 0.873[0.851–0.892] (p < 1.0e-12) and median AAPM MSD from 1.27[1.47–1.16] to 1.22[1.36–1.11] mm (p < 1.0e-6). Non-curated conditions benefited more from ensembles, as median[IQR] DSCs improved by an absolute 1.0/0.4 % to 0.871[0.848–0.889]/0.877[0.857–0.900] for non-curated/curated conditions, respectively. Ensembles improved DSC across all test/validation sets. RRSS results and training times are supplemented (SMB.2–3).

### 3.5 Curation error analysis

Quality scores (1–5) of curated cases (N = 50) were (6 %-26 %-48 %-18 %-2%). With score <4 as reference, curation specificity was estimated at 80 %. Curation agreement was 78 % (0 %: anti-agreement; 20 %: completely random (=curation rate); 100 %: perfect agreement among all iterations). Among 50 reviewed cases, 40 contained unacceptable shortcomings, and one contained no substantial shortcomings.

### 3.6 SMG curation

Acceptable SMG curation required 40 epochs. In-distribution true-performance DSCs significantly (p < 1.0e-3) from 89.3 % to 89.7 %. Of curated cases (N = 72), quality score occurrence rates were (20 %-40 %–23 %-13 %-4%), resulting in 83 % specificity.

## 4 Discussion

We introduced an easy-to-implement auto-curation approach which improved PG auto-segmentation performance. For this, we manually corrupted clean training segmentations and quantified performance loss: when 30 % of reference segmentations were corrupted, auto-segmentation performance decreased by ∼2.5 % absolute DSC. Our auto-curation removed 98 % of corrupted data and recovered the majority (92.1 %) of performance lost (Fig. 3). In real-world data (unknown corruption/noise extent), DSC improved 1.9 % at maximum (20 % curation) in independent, out-of-training-distribution data. Visual inspection showed improved consistency, particularly in medial/anterior lobes (Fig. 5). Since these results were constituted ad-hoc, they may potentially be overestimated, though repeated analyses indicate that auto-segmentation reliably benefits from auto-curation.

CNN performances for PG auto-segmentation on CT show typical median(range) DSCs of 0.85[0.82–0.88] [29], [30], [31], [32], [33], [34], and inter-rater variability of 0.76 [32]. With DSC = 0.87, our non-curated (apparent) results reach this upper range. Additionally, curation improved generalizability, without deteriorating this result. In corruption simulations, true performance suffered already with little corruption, and model deterioration from 30/15 % corruption was estimated at 2.5/1.8 % absolute DSC loss, while a relative recovery by curation was estimated at 1.5 %. Considering curated datasets contained 30 % fewer training samples, this recovery was high. In clinical experiments, curation was 78 % consistent. Considering that only one of reviewed cases showed both unusual anatomy (very small parotid), and no problems in the segmentation, only this case was wrongfully removed and curation showed high specificity with good consistency. Choosing lower curation rates, reviewing (and correcting) curated cases could effectuate more substantial benefits from minimal time investment.

The limited curation efficacy in the true-performance clinical compared to corrupted data may be due to dataset size: with limited training samples, CNNs may suffer more from sub-optimal segmentations. This is supported by repeated subsampled clinical data (Fig. 4, green; SMB.2), where curation slightly but consistently (across multiple curation fractions and iterations) improved both clinical true and apparent performances and by corruption experiments (Fig. 2), where small amounts of segmentation noise already impacted true performance.

DSC by itself is of limited value for CNN contour evaluation, as it can be insensitive to fine but clinically relevant details [35], [36], [37]. Complementarily, works suggest using distance-based metrics [38], [39]. Therefore, auto-curation was also evaluated using distance-based metrics. Since the used loss [40] did not involve distance-based information, observed effects in HD/MSD were indirect. Interestingly, where DSC did not improve among true- and apparent-performance data, slight but consistent improvements were observed in HD/MSD across multiple curation fractions (Fig. 4, purple). Though this effect was smaller in repeated experiments (Fig. 6), we expect that using distance-based losses and including HD/MSD as the curation basis could lead to more convincing improvements among these metrics.

The clinical relevance of an estimated 1–2 % DSC improvement by curation may be limited [11], [41], [42], and geometric measures (DSC/HD/MSD) had little predictive value with respect to dose coverage on target volumes and OARs [43]. Since here, the PG was used as a paradigm for studying curation, it could serve as a stepping-stone for investigating the utility of curation approaches for other situations where imperfect segmentations are inherent to the acquisition context (e.g., radiotherapy target [44]). SMG curation showed promising specificity, but additional experiments are needed to confirm the benefit in external data. Additionally, we observed that auto-curation removed structures mistakenly saved under wrong names, which occurs in clinical practice.

When considering auto-curation for other data, one important parameter is the amount of data to remove (curation rate). Although auto-curation results in partial exploration of training data, there exists a trade-off between removing poor labels (and difficult but potentially informative cases), and keeping training samples. Given the excellent sensitivity in simulations (>95 %), the removed amount should match the number of corrupted segmentations present in the training set for optimal effects. Although this is typically unknown clinically, we observed the highest overall performance gain at curation rates of 20 %, and removing less data still improved performance. Therefore, considering the high sensitivity in simulations, we recommend 15 % curation as a conservative middle ground for reliable but safe performance enhancement in clinical data. Alternatively, curating data up to a predefined DSC may be more robust for other datasets. Another important parameter is the curation time (epoch). For other datasets, this should be optimized for dataset size, quality and/or task complexity. For example, radiotherapy target segmentation may require more training for effective curation.

Typical radiotherapy segmentation datasets contain noise [1], [2], [3], [6]. Visual inspection of low-DSC cases from in-house datasets showed that these data contained several contouring imperfections, which were partly systematic (missing anterior/medial lobe; including skin/earlobe/bone/air) and partly random (stochastic deviations), resulting in different true and apparent performances. Practically, apparent performance observed in typical auto-segmentation data is not purely reflective of performance in correct cases. Using noisy training segmentations hurts performance on clean data, confirming that curation effects can be masked in typical validation data (Fig. 2, Fig. 3.3–3.7). RTCC experiments suggest that this is no issue if training datasets are sufficiently large, as both true and apparent (in-distribution) performances were uninfluenced by curation but generalized considerably better after curation (Fig. 4; AAPM RTCC DSC). However, when curating without clean clinical validation sets, benefits may be overestimated if datasets are smaller (Fig. 4; apparent RRSS).

Simulations showed curation benefits are largely masked in typical radiotherapy segmentation datasets (Fig. 3.4–3.5). True CNN performance benefited substantially from curation in simulations, whereas this was less prevalent in small clinical datasets and absent in RTCC. Despite careful matching of simulated corruptions to represent the severity of imperfections observed in clinical data, possibly, imperfections are smaller in clinical data. Most improvement was seen in AAPM, suggesting that in-house and external distributions differ considerably, and that curation helps accommodating distribution shifts. Additionally, with increasingly data-hungry algorithms (e.g. transformer and diffusion networks), future deep learning applications may involve centralized (anonymized) datasets for larger-scale model training and sharing among clinics. Therefore, as curation reduces institute-specific biases, auto-curation would be suitable as a precaution for auto-cleaning datasets at risk of substantial segmentation bias.

This study has limitations. First, in-house clinical datasets are based on subjective quality measures. It is challenging to guarantee “perfect” datasets, as experts do not always agree on segmentations. To mitigate this, two specialists carefully selected validation cases. Second, our data were obtained using one scanner, limiting generalizability. Regardless, AAPM segmentation performance resembled in-house performances, indicating that good generalizability was reached. Lastly, although inspired by clinically encountered problems (see SMB.1), synthetic erosions and dilations may have resulted in unnatural segmentation corruptions, which may impair how corruption experiments translate to clinical data.

Radiotherapy datasets naturally contain noisy segmentations, which degrade CNN performance. Auto-curation by removing low-DSC samples early during training can partially undo degradations that arise from bias and increase model generalizability, likely driven by a reduction in faulty reference segmentations that undermine stochastic gradient descent. Considering the adequate sensitivity and notable simplicity, we recommend applying auto-curation to network training when using real-world contours, where substantial bias is expected, to improve performance without needing to manually review training data.

## Funding Statement

This work has been funded by a research grant from Varian Medical Systems. O.J.G. is funded by the Dutch cancer society (KWF) grant number KWF-UVA 2017.10873.

## Declaration of competing interests

B.J.S. and W.F.A.R.V. declare honoraria and travel expenses with Varian Medical Systems, V.I.J.S. is funded by a research grant from Varian Medical Systems. Since May 2023, W.F.A.R.V is an employee of Varian Medical Systems. The department of radiation oncology of Amsterdam UMC has received a grant from Varian Medical Systems for this work.
