# Supplementary material for: Impact of annotation imperfections and auto-curation for deep learning-based organ-at-risk segmentation
Source: Phys Imaging Radiat Oncol. 2024 Dec 4;32:100684. doi: 10.1016/j.phro.2024.100684 (PMC11667007; doi:10.1016/j.phro.2024.100684)
Supplement: Supplementary Data 1 [file mmc1.docx]

**(A) Supplementary methods**

**A.1 Data pre-processing**

In-plane acquisition resolutions were (0.80-1.27) mm, with a median[interquartile range (IQR)] of 0.98[0.97-1.02]. The slice thickness was 2.5 mm in 96% of cases and 1.25 mm for the remaining cases. In-plane acquisition dimensions were 512x512x(98-581). All patient planning CTs and segmentations were interpolated to a 1x1x1.25 mm^3^ voxel spacing by 3^rd^-order spline and nearest-neighbor interpolation, respectively. This spacing was chosen to as an optimal trade-off for resolution (to allow for better erosions in corruption simulation experiments) while minimizing interpolations. To accommodate dose calculation, radiotherapy CTs contain a large field-of-view (FOV). To reduce the memory required for our networks, we wrote an automated algorithm that cropped the CT (176x192x176 voxels) to only contain the relevant regions of the CT necessary for segmentation. As patients were placed in the scanner with varying native positions and neck flexions during treatment, patients’ heads showed large positional variations in CT DICOMs. Using the same image coordinates for constructing the field-of-view (FOV) for all patients would cause parotid gland to fall outside of it for some. We ensured that for all patients, both parotid glands were encompassed in the FOV by algorithmically finding the 6 borders for each patient individually (Fig. SMA.1). First, the couch was identified by selecting the axial slice where the derivative of the slice-averaged Hounsfield Units (Hus) exceeded one standard deviation (Fig. SMA.1a; red arrow), after which it was removed from the planning CT by casting all couch slices to air HUs (-1000). Second, the cranial end slice of the FOV was detected. To do this, the CT was first window-leveled to between -200 and 300, which was previously reported to serve as a good window leveling for most head and neck automated segmentation tasks[1]. Then, a cranial reference point was detected as the point in the face area where the HUs summed along the axial axis was maximal (Fig. SMA.1b; blue arrow). From this, the cranial border was selected 30 slices (=3.75 cm) above the cranial reference point, and the caudal border was taken as the 176 slices (=22 cm) below that. There was some variability in the cranial reference point, but since the axial FOV was sufficiently large, this did not lead to problems. Next, the central sagittal (reference) slice was estimated by the median slice for which the mean HUs exceeded 90% of the maximum (Fig. SMA.1c; blue arrow). The lateral FOV borders were then found by the slices ±96 slices from the selected central slice. Next, an anterior reference slice was found where the mean HU of center slice exceeded -950 (Fig. SMA.1d; blue arrow). This means that, in cases where a thermoplastic mask was used, the reference was more anterior than in cases where it was not used. From this reference, the anterior border was 1 cm (10 voxels) posterior to the reference, and the posterior border was 176 slices from the anterior border. The FOV was then laterally divided in half (Fig. SMA.1e) to obtain the (unilateral) images used for training. CT HUs were then rescaled between [0-1]. Finally, images were checked such that all parotid gland voxels were contained well within the selected FOV and corrected where necessary.


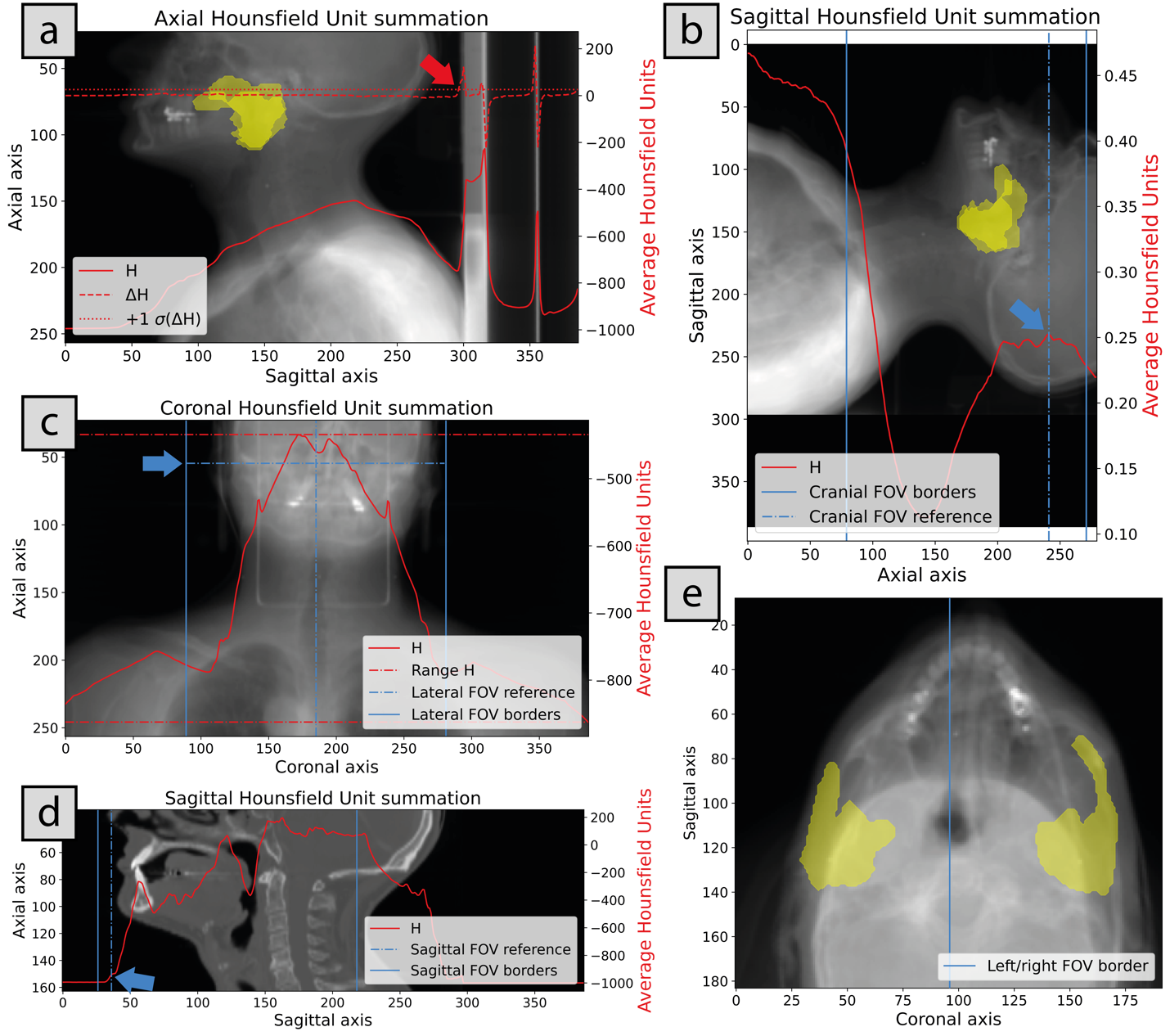


***Figure SMA.1*** ***COLOR***: *Pre-processing overview. In* sub-figures A-D, function H indicates the average Hounsfield Units (HUs) over the slice depicted on the x-axis. (a) Projection of CT HUs along the coronal axis. The couch was detected as the slice where the derivative of H exceeded one standard deviation (red arrow); (b) Projection CT in (a), but with the couch removed and rotated 90 degrees in the sagittal plane. The uppermost slice of the head was detected as the point in the face area where the HUs summed along the axial axis was maximal (blue arrow); (c) Projection of CT HUs along the sagittal axis. The central sagittal reference was estimated by the median slice for which H exceeded 90% of its maximum (blue arrow). This method was chosen because it was observed to make selection of the central slice more robust to rotations of the neck. Lateral FOV borders (blue solid lines) were then determined by the ±96 slices with respect to the reference central midpoint slice. (d) Anterior reference was selected where the mean HU of the center slice exceeded -950 (blue arrow); (E) Example of the selected 176x192x176 FOV. The yellow contours indicate projections of the parotid gland voxels in the clinical reference segmentation.

**A.2 Preliminary experiments**

Two important parameters for curation are the epoch of convergence (i.e. the number of epochs trained for fully training the CNN) and the curation epoch (i.e. the epoch chosen to perform the curation operation. As the curation epoch directly influences the training distribution in the first step of the curation operation and depends on the epoch of convergence, it was important that that these epochs were appropriately chosen. To do this, training and validation losses were monitored during the optimization of 5 models with different initializations for each experiment line (Tab. SMA.3, A-D). The point of convergence was defined as the point where the mean validation loss over all models plateaued. We found that this point happened only at very late stages of training, suggesting that the performed augmentations and learning rates were effective in preventing overfit. The curation epoch was chosen as the epoch where, on average, models achieved acceptable performance, while minimizing training time. In practice, this point was reached at 25% of the epoch of convergence, which was 40 when the number of training cases exceeded 1000 (Experiment series A D(3-4)) and 100 when the number of training cases were 100 (Experiment series B, C, D(1,2)). If curation time is an issue, we believe that this curation method may work at lower than 25% of total epochs, but this was chosen to be on the safe side. For the submandibular gland, it was observed that 100 training epochs were needed for full training and 40 training epochs were needed for optimal curation efficacies. Since errors and inconsistencies were estimated to be less severe in SMG segmentation data, a lower curation fraction of 5% was selected for post-hoc curation experiments.

**A.3 Experimental outline (Parotid gland)**

***Table SMA.3*:** Overview of the experimental outline of this study. Q1 indicates the highest Dice similarity coefficient quartile of RTCC, resulting from the cross-validation (A). Numbers indicate the number of samples. The data source indicates where the training data is sampled from. Abbreviations: CV: cross-validation; RRSS: repeated random sub-sampling; RTCC: radiotherapy clinical cohort.

| **Parotid gland** | | | | | | | | |
| --- | --- | --- | --- | --- | --- | --- | --- | --- |
| **Series** | **A** | **B** | | **C** | | | **D** | |
| **Experiment** | **CV** | **Corruption impact** | | **Corruption curation** | | | **Clinical curation** | |
| Sub-indicator | - | 1 | 2 | 1-3 | 4-5 | 6-7 | 1-2 | 3-4 |
| Data source | RTCC | Q1 | Q1 | Q1 | Q1 | Q1 | RRSS | RTCC |
| Corruption | - | Systematic | Random | - | Systematic | Random | - | - |
| Train | 1365 | 100 | 100 | 100 | 100 | 100 | 100 | 1750 |
| Validation | 175 | 20 | 20 | 20 | 20 | 20 | 125 | 125 |
| Internal test | 385 | 40 | 40 | 40 | 40 | 40 | 50 | 50 |
| External test | - | - | - | - | - | - | 96 | 96 |

**A.4 Curation translation to other datasets**

***Table SMA.4*:** Overview of the data used for curation experiment using submandibular gland data. For curation, the external data was included in the training data, to obtain a training set of 1435 individual submandibular glands.

| **Submandibular gland (post-hoc) clinical curation data** | | | |
| --- | --- | --- | --- |
| Train | Validation | Internal test | External test |
| 1363 | 171 | 170 | 72 |

**A.5 Model training**

Model training was done on four NVIDIA-GeForce GTX 2080Ti GPUs, with 11GB GPU RAM, 64GB system RAM and an Intel(R) Core(TM) i9-9900KF CPU@3.6GHz processor. The GPU-version of PyTorch (Version 1.13) with Cuda 11.8 and Python (Version 3.10.0) were used. Weights were initialized using Pytorch’s standard initialization method and models were trained using the ADAM optimizer[2], using a combination of Dice similarity coefficient (DSC)[3] and focal loss[4] to guide model optimization[5]:

$L_{UNet}=L_{DSC}+ L_{foc}$ *(Eq. 4 – 1)*

where focal loss accommodates the size-dependency of DSC loss and balances foreground and background voxels^48^. Here, A and B denote the predicted and clinical segmentation binary sets, respectively. Models were trained using standard hyper-parameter settings, with β_1_ = 0.9, β_2_ = 0.999 and ε = 1×10^−7^, a batch size of 1 and an initial learning rate of 0.001. To mitigate the divergence of model weights at later stages of training, Pytorch’s cosine annealing learning rate[6] was used without warm restarts, with a minimum (final) learning rate of 1×10^−5^. Per experiment series, we heuristically chose the number of epochs required to fully train the baseline (without curation) CNN, as described in the experimental outline, and the learning rate, which depended directly on the number of epochs. As samples were removed during curation, number of epochs was adjusted such that an equal amount of data was seen per experiment, to ensure fair comparisons. In each experiment series, the validation loss was used to select the model used for evaluation. An overview of the exact settings used for each model and how curation affected training epochs can be viewed in Tab. SMA.5.

***Table SMA.5*:** Overview of the settings used for model training throughout this study. Abbreviations: RRSS: repeated random sub-sampling; RTCC: radiotherapy clinical cohort.

| **Corruption and curation of simulated segmentation corruptions and RRSS (experiment series B, C, D3-4)** | | | | | | | | | | | |
| --- | --- | --- | --- | --- | --- | --- | --- | --- | --- | --- | --- |
| Curation  fraction (R) | 0 | 1 | 5 | 10 | 15 | 20 | 25 | 30 | 50 |  | 90 |
| Epochs | 100 | 101 | 105 | 111 | 118 | 125 | 133 | 143 | 200 |  | 1000 |
| Samples per  epoch | 100 | 99 | 95 | 90 | 85 | 80 | 75 | 70 | 50 |  | 10 |
| Total | 10.000 | 9.999 | 9.975 | 9.990 | 10.030 | 10.000 | 9.975 | 10.010 | 10.000 |  | 10.000 |
|  |  |  |  |  |  |  |  |  |  |  |  |
| **Baseline cross-validation and curation of RTCC (experiment series A, D1-2)** | | | | | | | | | | | |
| Curation  fraction (R) | 0 |  |  | 10 |  | 20 |  | 30 | 50 | 75 | 90 |
| Epochs | 40 |  |  | 44 |  | 50 |  | 57 | 80 | 160 | 400 |
| Samples per  epoch | 1.750 |  |  | 1.575 |  | 1.400 |  | 1.225 | 875 | 437 | 175 |
| Total | 70.000 |  |  | 69.300 |  | 70.000 |  | 69.825 | 70.000 | 69.920 | 70.000 |

Data augmentations were performed on-the-fly, using Medical Open Network for AI (MONAI), an established, open-source, PyTorch framework[7]. Modes of augmentation were: (1) rotations (p: 0.2) of up to 15 degrees using bilinear and nearest-neighbour interpolations for CT and contours, respectively. (2) zooms (p: 0.2) between 0.9 and 1.1 scaling factor; (3) left/right flipping (p: 0.5); (4) intensity shifts (p: 0.2) with an offset of 0.1. Augmentations were turned off at test time.

**(B) Supplementary results**

**B.1. Clinically observed segmentation errors**


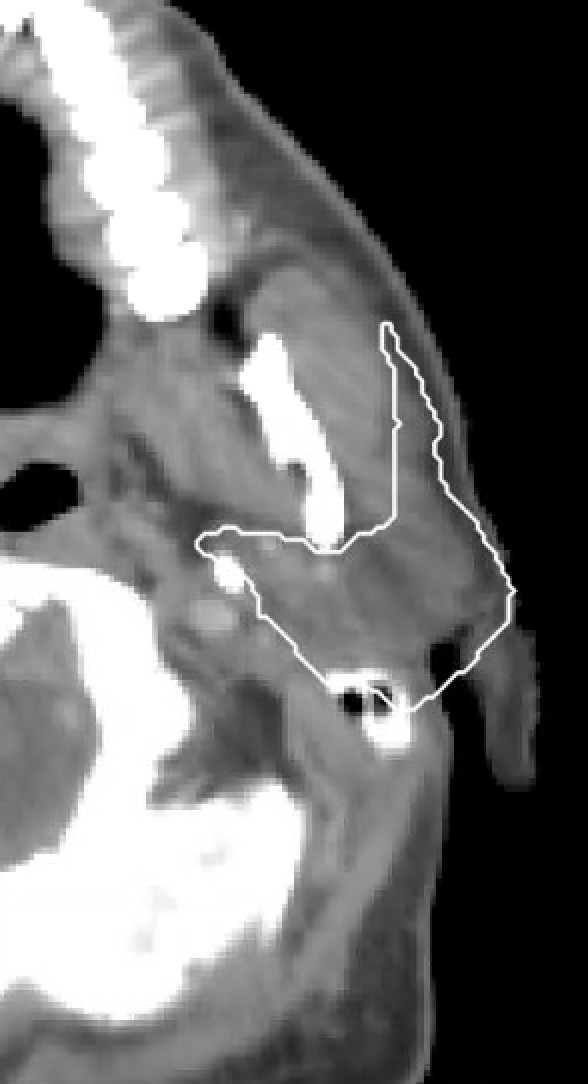

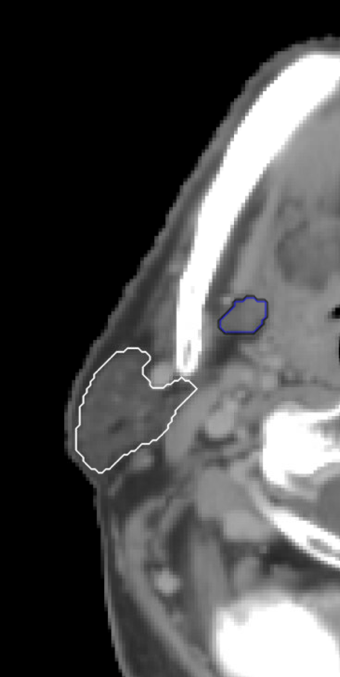

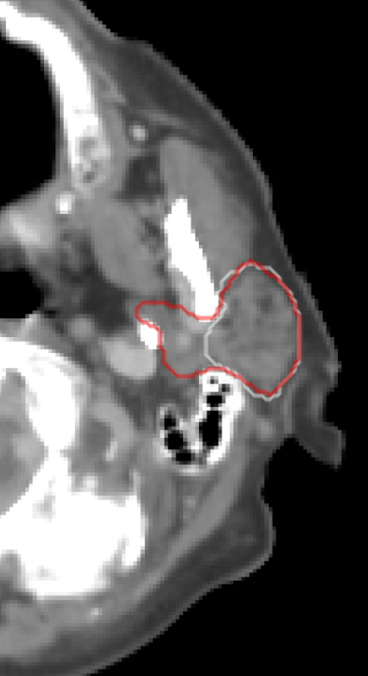

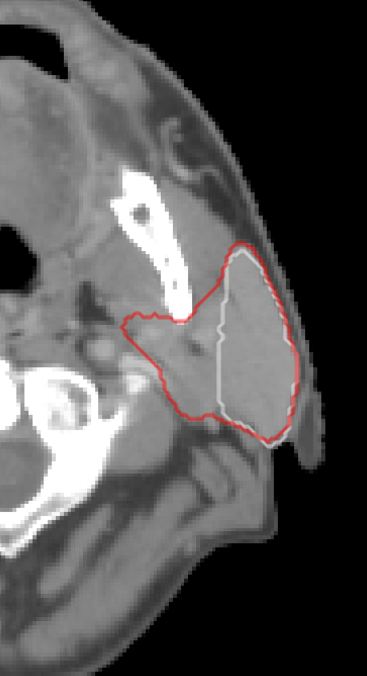


***Figure SMB.1 COLOR*** *Inaccurate segmentation examples*. Four examples of cases that were removed by the curation operation. From left to right: (1-2) large portions of medial parotid gland tissue were missing in the ground truth (white) segmentation, versus the deep learning predicted parotid gland contour (red); (3) example of a parotid gland reference contour (white) that was mistakenly saved as submandibular gland, versus the deep learning predicted submandibular gland contour (blue). (4) Example of a parotid gland reference segmentation (white) where bone and air / ear tissue was included.

**B.2 Repeated random sub-sampling (RRSS)**

When datasets were small, apparent median DSC slightly but consistently improved for curation fractions 5-30% (Fig. 4). Curation rates of 20% provided the largest improvement across all evaluation data and metrics. Curation generally improved apparent DSC more than true DSC. True HD deteriorated over curation fractions, while apparent HD and true and apparent MSD, varied around the non-curation baseline below 30% curation. Curation was consistently better than random sample removal. Weak (R^2^<0.4) correlations were found between curation effect and sampled distributions.

**B.3 Training times**

With 100 training samples per epoch, training times (mean±standard deviation) of 75 models was 128.5±0.66 per model. With 1750 training samples per epoch, training times of 32 models were 22.7±0.85 hours per model.

**References**

1. van Rooij W, Dahele M, Brandao HR, Delaney AR, Slotman BJ, Verbakel WFAR. Deep Learning-Based Delineation of Head and Neck Organs at Risk: Geometric and Dosimetric Evaluation. *Int J Radiat Oncol Biol Phys* 2019;3:104:677–684. doi: <https://doi.org/10.1016/j.ijrobp.2019.02.040>.
2. Kingma DP, Ba J. Adam: A Method for Stochastic Optimization. in *3rd ICLR* 2015. doi: https://doi.org/[10.48550/arXiv.1412.6980](https://doi.org/10.48550/arXiv.1412.6980).
3. Sudre CH, Li W, Vercauteren T, Ourselin S Cardoso MJ. Generalised Dice overlap as a deep learning loss function for highly unbalanced segmentations. LNCS 2017:10553. doi: <https://doi.org/10.1007/978-3-319-67558-9_28>.
4. Lin TY, Goyal P, Girshick R, He K Dollár P. Focal Loss for Dense Object Detection. In *IEEE PAMI* 2017. doi: https://doi.org/[10.48550/arXiv.1708.02002](https://doi.org/10.48550/arXiv.1708.02002).
5. Zhu W, Huang Y, Zeng L, *et al.* AnatomyNet: Deep learning for fast and fully automated whole-volume segmentation of head and neck anatomy. *Med Phys* 2019. doi: <https://doi.org/10.1002/mp.13300>.
6. Loshchilov I, Hutter F. SGDR: Stochastic gradient descent with warm restarts. in *5^th^ ICLR 2017 - Conference Track Proceedings* 2017. doi: <https://doi.org/10.48550/arXiv.1608.0398>
7. Project MONAI — MONAI 0 Documentation. https://docs.monai.io/en/stable/.
